# Supplementary material for: RNF43 truncations trap CK1 to drive niche‐independent self‐renewal in cancer
Source: EMBO J. 2020 Aug 10;39(18):e103932. doi: 10.15252/embj.2019103932 (PMC7503102; doi:10.15252/embj.2019103932)
Supplement: Supplementary file 1 — Appendix [file EMBJ-39-e103932-s001.pdf]

RNF43 truncations trap CK1 to drive niche-independent self-renewal in cancer

Spit et al. 2020

Appendix

Table of contents

**Appendix Figure S1**	**Page 1**
**Appendix Figure S2**	**Page 2**
**Appendix Figure S3**	**Page 3**
**Appendix Figure S4**	**Page 4**
**Appendix Table S1**	**Page 5**
**Appendix Table S2**	**Page 6**
**Appendix Table S3**	**Page 7**
**Appendix Table S4**	**Page 8**

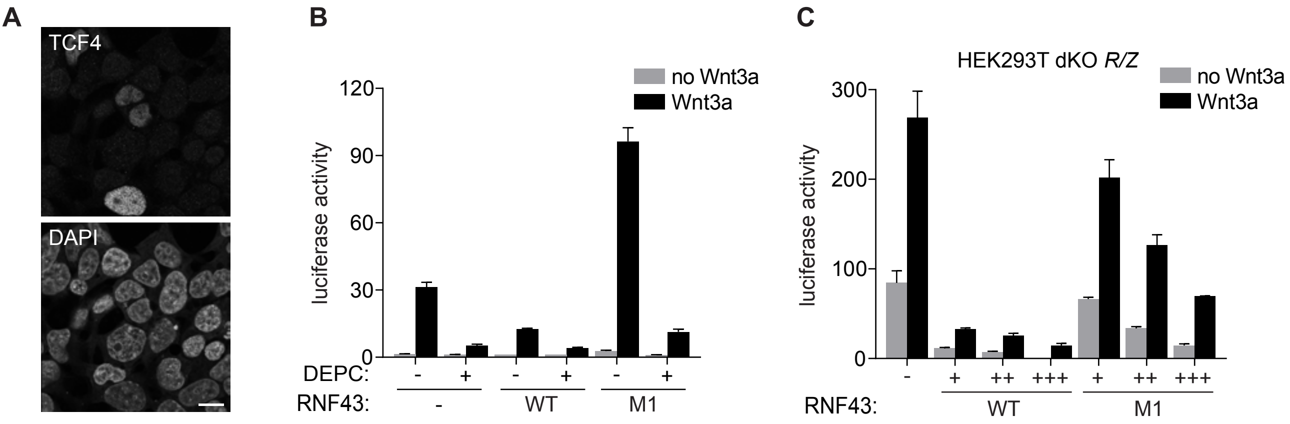

Appendix Fig S1. TCF4 expression and the effects of RNF43 ligase dead mutant

A. Microscopy analysis of HEK293T cells expressing ΔN-TCF4 revealed by anti-TCF4 for the experiment shown in Fig 2E. Scale bar represents 10 μM.

B. β-catenin-mediated reporter activity in HEK293T cells co-expressing Dvl-DEPC with RNF43 WT or the ligase dead mutant (M1) in the presence and absence of Wnt3a-CM. Average β-catenin-mediated reporter activities ±s.d. in n = 2 independent wells are shown.

C. β-catenin-mediated reporter activity in HEK293T double knock out (dKO) *RNF43/ZNRF3* (*R/Z*) cells expressing increasing amounts of RNF43 WT or the ligase dead mutant (M1) in the presence and absence of Wnt3a-CM. Average β-catenin-mediated reporter activities ±s.d. in n = 2 independent wells are shown.

Appendix Figure S2; Spit et al, 2020

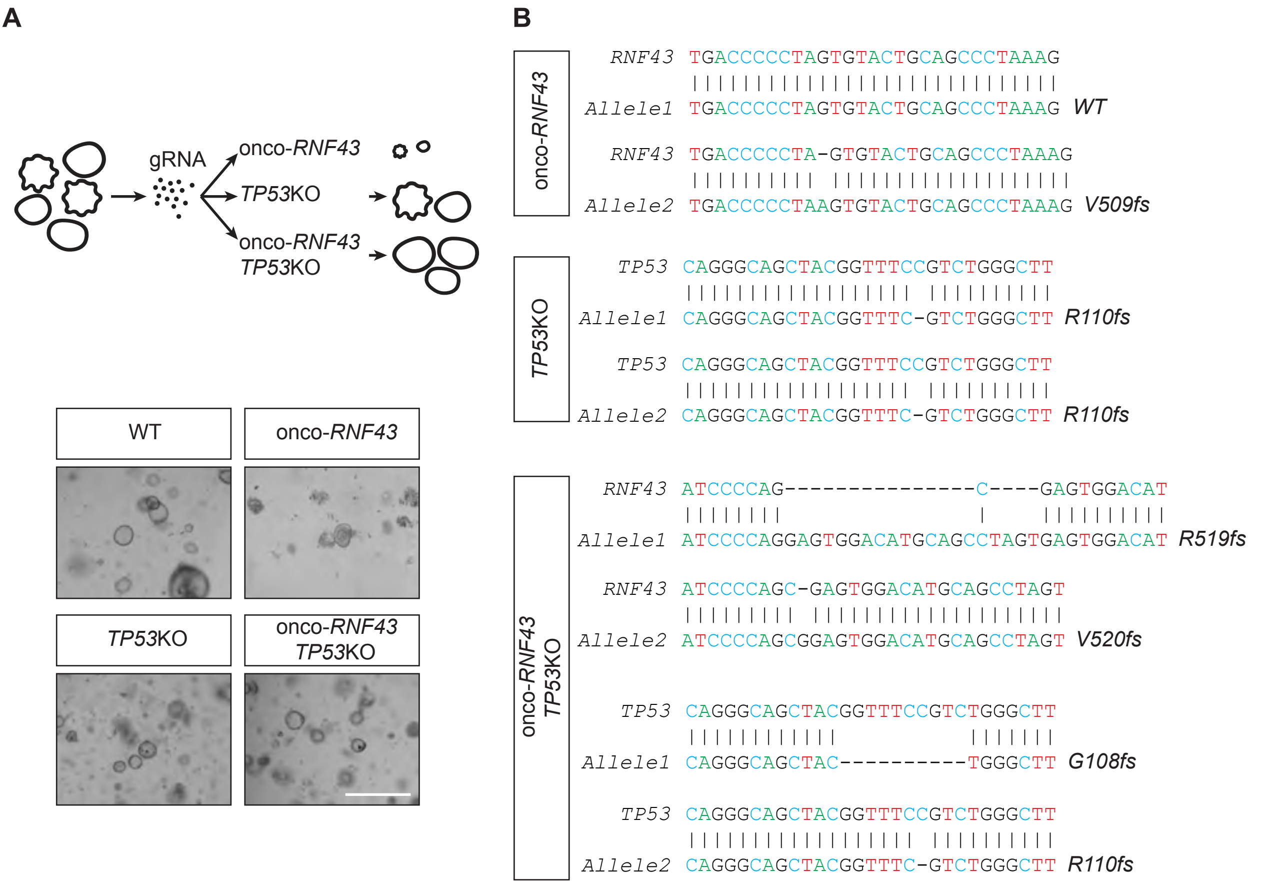

Appendix Fig S2. Ablation of *TP53* is permissive for growth of onco-RNF43-expressing organoids

A. Strategy to engineer the indicated human colon organoid lines. Bright-field microscopy pictures of WT, mono-allelic onco-*RNF43*, *TP53*KO and onco-*RNF43*/*TP53*KO human colon organoids. Scale bar represent 400 μm.

B. Sanger sequencing confirmation of onco-*RNF43*, *TP53*KO and onco-*RNF43*/*TP53*KO mutations in human colon organoids. Sequencing results for each allele compared to the wild type alleles are shown. The top line always represents the WT sequence (either TP53 or RNF43). The bottom lines represent the aquired sequences after CRISPR/Cas9 modulation, including various deletions resulting in frameshift mutants of both *TP53* and *RNF43*.

Appendix Figure S3; Spit et al, 2020

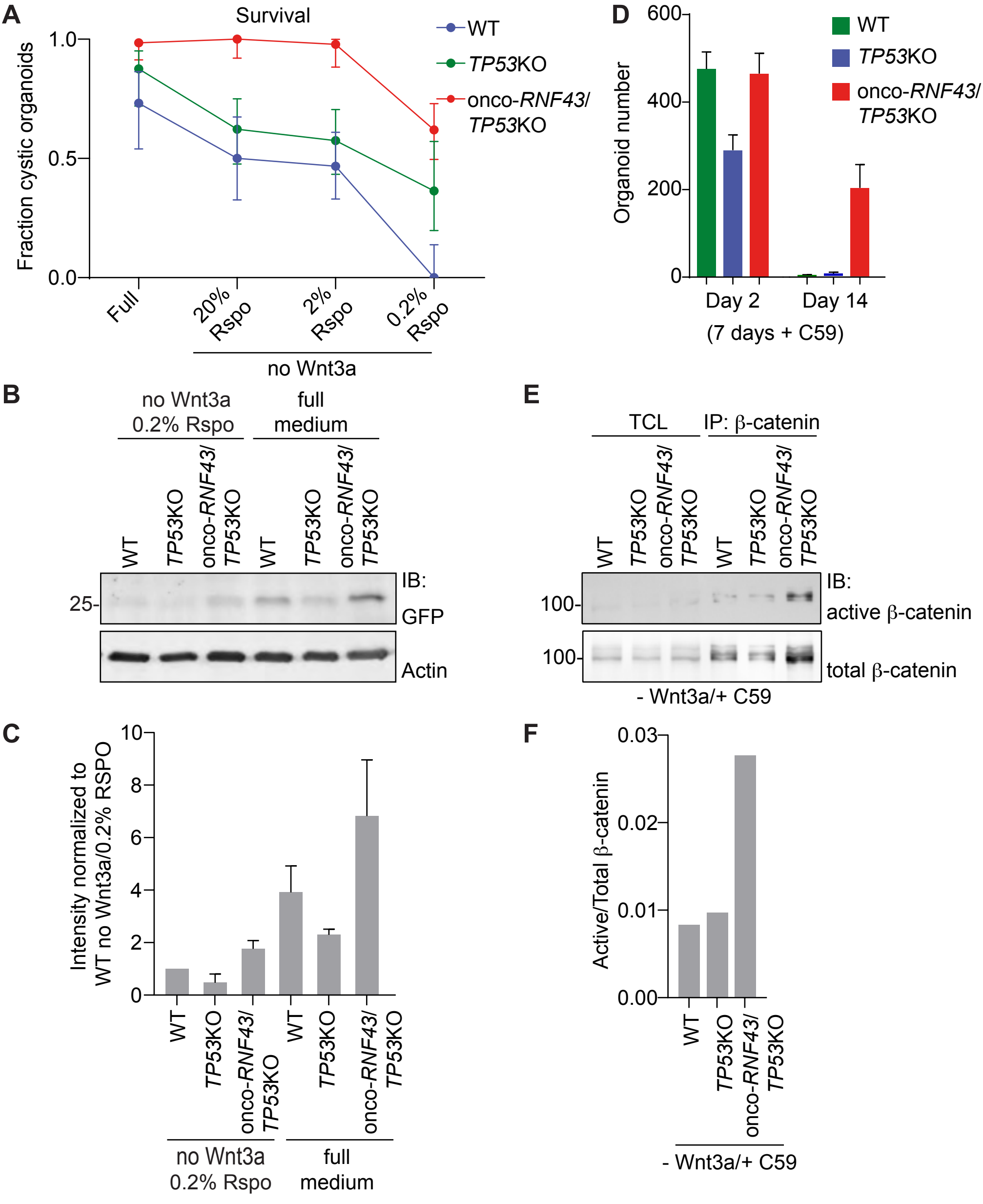

Appendix Fig S3. Onco-*RNF43/TP53*KO organoids are resistant to low levels of Rspo and display higher levels of Wnt signaling

A. Fraction of WT, *TP53*KO and onco-*RNF43*/*TP53*KO human colon organoids that presented a cystic, proliferative morphology in the conditions described in Fig 4A. Error bars represent 95% confidence interval (Wilson/Brown test). n = 24-63 organoids per condition.

B. Western blot analysis of WT, *TP53*KO and onco-*RNF43*/*TP53*KO human colon organoid lines grown in two different media and transduced with the TOP-GFP reporter. Organoids were lyzed 6 days after splitting and the indicated antibodies were used for detection.

C. Quantification of the Western blot presented in (B). GFP levels were quantified and normalized to actin. Graph represents the mean normalized to no Wnt3a/0.2% Rspo medium ±s.d. of 2 independent experiments.

D. Number of WT, *TP53*KO and *onco-RNF43*/*TP53*KO organoids at day 2 and day 14. Organoids were treated for 7 days with PORCN inhibitor C59 (1μM) after which the medium was replaced and organoids were split at day 9. Error bars represent ±s.d. of the mean\ of n = 3 experiments.

E. Western blot analysis of an endogenous β-catenin immunoprecipitation in WT, *TP53*KO and onco-*RNF43*/*TP53*KO human colon organoid lysates. Organoids were grown in full medium for 5 days and 24h before lysis placed in medium with no Wnt3a/no Rspo supplemented with PORCN inhibitor C59 (1 μM). Both total levels of β-catenin and active, non-phosphorylated β-catenin were detected.

F. Quantification of the Western blot presented in (E). Active, non-phosphorylated β-catenin levels were quantified and normalized to total levels of β-catenin. Graph represents the mean.

Appendix Figure S4; Spit et al, 2020

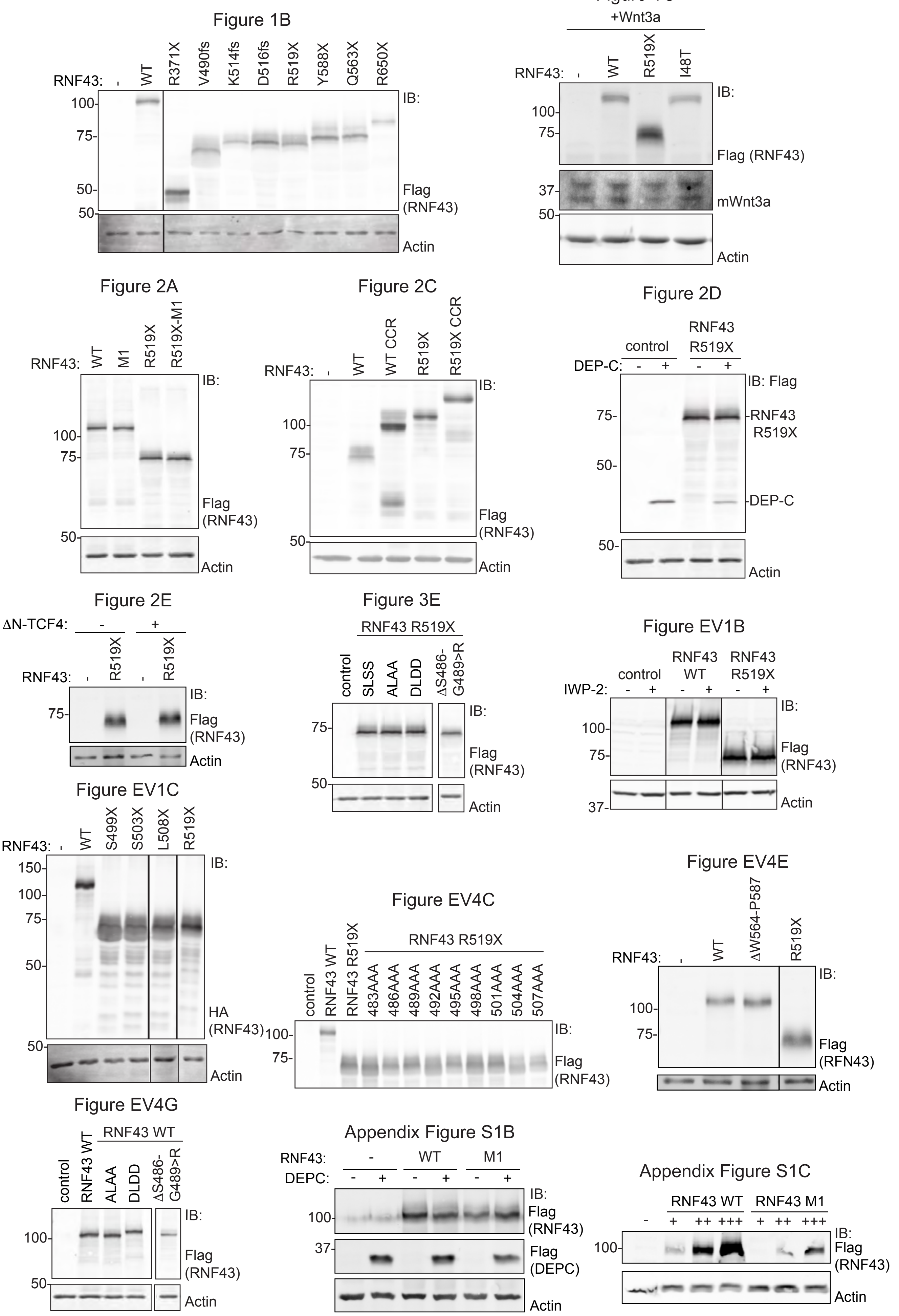

Appendix Fig S4
Western blots for the indicated β-catenin-mediated reporter assays.

Appendix Table S1. Onco-*RNF43* mutations in human tumors.

Study	Sample ID	Cancer Type	Amino acid change	Mutations per sample	Mutations in senescence genes
Breast Invasive Carcinoma (TCGA, Nature 2012)	TCGA-AN-A04D-01	Invasive Breast Carcinoma	K514Sfs*9	71	RB1 (deep deletion)
TCGA data for Esophagus-Stomach Cancers (TCGA, Nature 2017)	TCGA-BR-8081-01	Stomach Adenocarcinoma	D516Gfs*10	946	TP53, ATM
Uterine Corpus Endometrial Carcinoma (TCGA, PanCancer Atlas)	TCGA-D1-A2G0-01	Uterine Mixed Endometrial Carcinoma	D516Ifs*11	817	PTEN
Merged Cohort of LGG and GBM (TCGA, Cell 2016)	TCGA-HT-7690-01	Diffuse Glioma	R519*	15	TP53
Uterine Corpus Endometrial Carcinoma (TCGA, Provisional)	TCGA-AP-A059-01	Uterine Endometrioid Carcinoma	R519*	9247	TP53, ATM, ATR, RBL1, PTEN
Uterine Corpus Endometrial Carcinoma (TCGA, PanCancer Atlas)	TCGA-AX-A2HD-01	Uterine Endometrioid Carcinoma	R519*	7855	ATM, TP63, RB1, RBL1, ATR, PTEN
Uterine Corpus Endometrial Carcinoma (TCGA, PanCancer Atlas)	TCGA-EY-A549-01	Uterine Endometrioid Carcinoma	R519*	1321	TP63, ATR, RB1, PTEN
Colorectal Adenocarcinoma (DFCI, Cell Reports 2016)	coadread_dfci_2016_2945	Colorectal Adenocarcinoma	S525Lfs*172	131	TP53
MSK-IMPACT Clinical Sequencing Cohort (MSKCC, Nat Med 2017)	P-0007969-T01-IM5	Stomach Adenocarcinoma	T542Pfs*158	46	ATR
Prostate Adenocarcinoma (TCGA, Provisional)	TCGA-CH-5746-01	Prostate Adenocarcinoma	S546Qfs*50	32	RBL1 (deep deletion)
Pan-Lung Cancer (TCGA, Nat Genet 2016)	TCGA-L9-A7SV-01	Lung Adenocarcinoma	Y558*	1341	TP53
MSK-IMPACT Clinical Sequencing Cohort (MSKCC, Nat Med 2017)	P-0012041-T01-IM5	Pancreatic Adenocarcinoma	F562Sfs*138	8	TP53, ATM, RB1
Skin Cutaneous Melanoma (TCGA, Provisional)	TCGA-EE-A2GJ-06	Cutaneous Melanoma	Q563*	928	TP53

Appendix Table S2. List of Wnt destruction complex-associated proteins identified by BioID.

ID	Name	PD 1	CTRL 1	PD 2	CTRL 2	PD 3	CTRL 3
Q68DV7	E3 ubiquitin-protein ligase RNF43 OS=Homo sapiens GN=RNF43 PE=1 SV=1 - [RNF43_HUMAN]	68(68)	-	69(69)	-	73(73)	-
O14641	Segment polarity protein dishevelled homolog DVL-2 OS=Homo sapiens GN=DVL2 PE=1 SV=1 - [DVL2_HUMAN]	17(18)	-	26(27)	-	14(14)	-
Q92997	Segment polarity protein dishevelled homolog DVL-3 OS=Homo sapiens GN=DVL3 PE=1 SV=2 - [DVL3_HUMAN]	15(17)	-	17(19)	-	10(11)	-
O14640	Segment polarity protein dishevelled homolog DVL-1 OS=Homo sapiens GN=DVL1 PE=1 SV=2 - [DVL1_HUMAN]	12(13)	-	16(17)	-	14(15)	-
P25054	Adenomatous polyposis coli protein OS=Homo sapiens GN=APC PE=1 SV=2 - [APC_HUMAN]	26(26)	-	22(22)	-	17(17)	-
O15169	Axin-1 OS=Homo sapiens GN=AXIN1 PE=1 SV=2 - [AXIN1_HUMAN]	5(5)	-	9(9)	-	5(5)	-
P49674	Casein kinase I isoform epsilon OS=Homo sapiens GN=CSNK1E PE=1 SV=1 - [CK1E_HUMAN]	2(2)	-	3(4)	-	2(2)	-
P48729	Casein kinase I isoform alpha OS=Homo sapiens GN=CSNK1A1 PE=1 SV=2 - [CK1A_HUMAN]	2(2)	-	2(2)	-	2(2)	-

Number of identified unique peptides are shown. Total identified peptides are indicated between brackets. PD, pull down; CTRL, control

Appendix Table S3. Phosphopeptides identified in RNF43 WT upon stimulation with Wnt3a and Rspo1.

Phosphosites	# P	RNF43 WT		
		control	Wnt3a	Wnt3a/ Rspo1
S251	1	5.50	5.00	5.68
S325	1	5.76	5.22	6.25
S443, S446	2	4.57	ND	5.60
S444	1	5.11	5.13	5.93
S532	1	5.88	5.16	5.79
S593	1	7.71	7.18	7.97
S603, S611	2	7.02	6.84	7.47
S611	1	6.30	5.95	6.74

The table summarizes the intensities of the identified phosphopeptides in the Log_{10} scale. Areas of indicated phosphopeptides were normalized based on the median. Non-phosphorylated peptides were used for the second part of normalization.

Phosphosite: position of phosphosite in the RNF43 protein sequence
P: number of phosphorylations observed on the peptide for the specific peptide spectrum match (PSM)
ND: not detected

Appendix Table S4. Phosphopeptides identified in RNF43 WT and R519X upon expression of CK1.

Phosphosites	# P	RNF43 WT			RNF43 R519X		
		no kinase	CK1α	CK1ε	no kinase	CK1α	CK1ε
S241	1	ND	ND	ND	7.37	7.20	7.21
S241-T245	1	ND	ND	ND	6.67	6.44	6.76
T308	1	ND	ND	ND	6.93	7.07	7.21
T317	1	ND	ND	ND	6.80	7.04	7.16
S321	1	ND	ND	ND	8.54	8.30	8.60
S325	1	6.65	5.85	6.32	7.23	7.10	7.40
S323-S325	1	ND	ND	ND	7.49	7.48	7.82
S331	1	5.74	6.26	5.40	7.37	7.33	7.62
S443-S446	2	6.48	7.45	7.12	8.45	8.36	9.05
S446	1	7.31	7.64	7.49	8.61	8.61	9.03
S443, S444, S446	3	NQ	NQ	NQ	ND	NQ	ND
S499-S503	1	ND	ND	ND	7.00	7.08	7.58
S499-S503	2	ND	ND	ND	ND	6.14	6.28
S512	1	6.79	7.03	6.43	7.49	7.09	7.52
S532	1	8.52	8.63	8.33	ND	ND	ND
S535	1	6.47	6.83	6.31	ND	ND	ND
S532, S535	2	ND	7.37	ND	ND	ND	ND
S525-S535, T539	2	ND	6.74	ND	ND	ND	ND
S607-S611	1	7.50	7.85	7.13	ND	ND	ND
S611	1	6.19	6.60	6.13	ND	ND	ND

The table summarizes the intensities of the identified phosphopeptides in the Log_{10} scale. Areas of indicated phosphopeptides were normalized. Phosphopeptide standards added prior to TiO_2 fractionation were used for normalization. Non-phosphorylated peptides were used for the second part of normalization. Individual areas of the phosphopeptides were combined and presented as the total area of each phosphosite.

Phosphosite: position of phosphosite in the RNF43 protein sequence
Phosphosites separated by a dash: phosphorylation was detected and occurs on one (or more) amino acids within the listed range, but position cannot be determined with certainty
P: number of phosphorylations observed on the peptide for the specific peptide spectrum match (PSM)
ND: not detected
NQ: identified, but cannot be quantified
In grey: below detection limit, for quality only
