# Supplementary material for: RNF43 truncations trap CK1 to drive niche‐independent self‐renewal in cancer
Source: EMBO J. 2020 Aug 10;39(18):e103932. doi: 10.15252/embj.2019103932 (PMC7503102; doi:10.15252/embj.2019103932)
Supplement: Supplementary file 2 — Expanded View Figures PDF [file EMBJ-39-e103932-s002.pdf]

Expanded View Figures

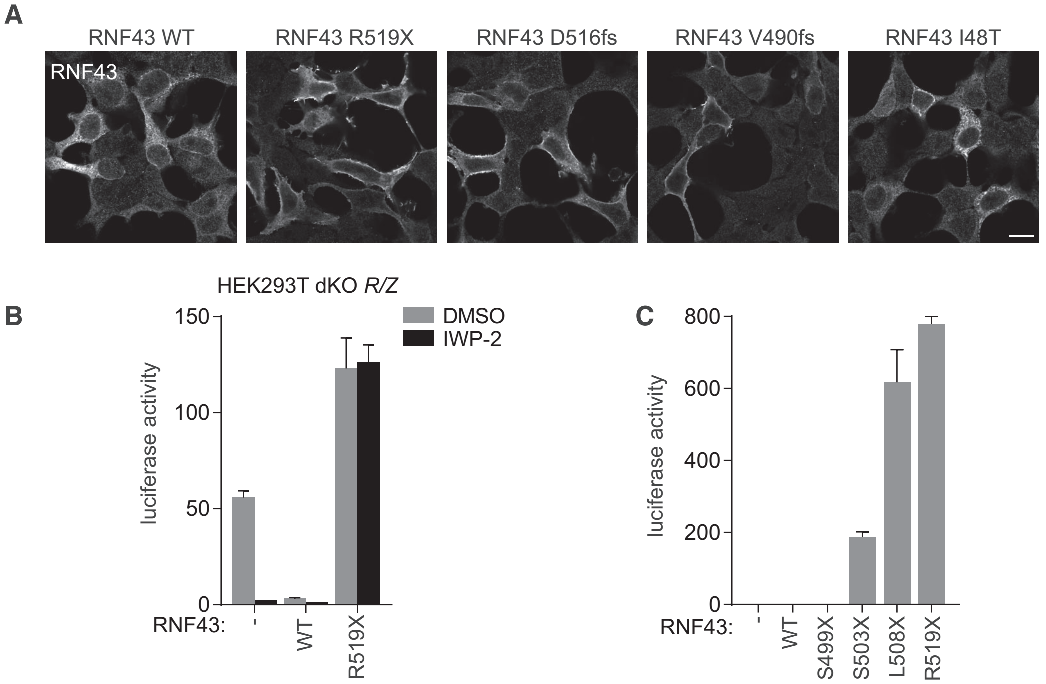

Figure EV1. Defining the region for oncogenic RNF43 truncations.

A RNF43 stainings of cells of the experiment shown in Fig 1E. Scale bar represents 10 μm.

B β-catenin-mediated reporter activity in HEK293T double knockout (dKO) *RNF43/ZNRF3* (*R/Z*) cells expressing RNF43 WT and oncogenic RNF43 (R519X) after treatment with DMSO or the PORCN inhibitor IWP-2 (5 μM; o/n). Average β-catenin-mediated reporter activities ± s.d. in n = 2 independent wells are shown.

C β-catenin-mediated reporter activity in HEK293T cells expressing the indicated RNF43 truncations in the absence of Wnt3a. Average β-catenin-mediated reporter activities ± s.d. in n = 2 independent wells are shown.

Data information: WT; wild-type.

© 2020 The Authors

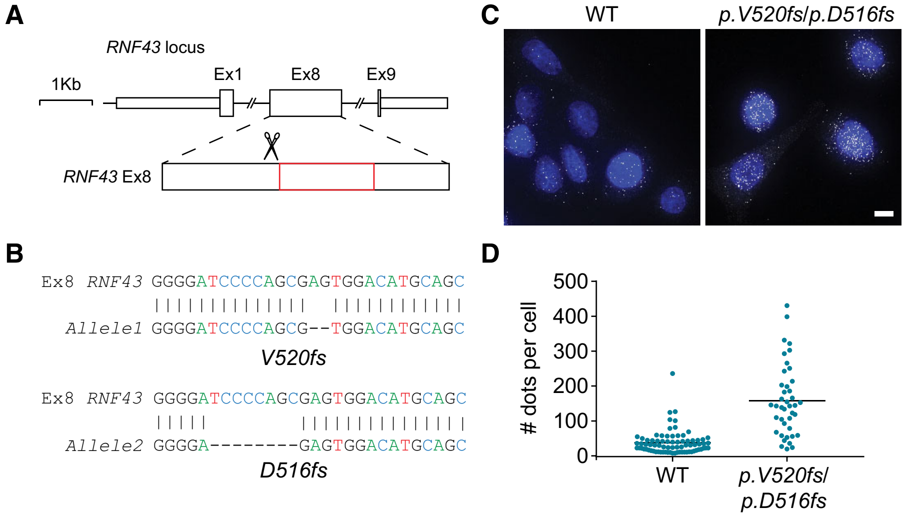

Figure EV2. Endogenous oncogenic RNF43 mRNA transcripts are stably expressed.

A Schematic representation of the targeted exon of human *RNF43*. Oncogenic region is indicated in red.

B Sanger sequencing of the PCR amplification products of the mutated *RNF43* alleles in SW480 cells. Sequencing results for each mutant allele compared to wild-type are shown. The top lines illustrate the wild-type *RNF43* sequence of nucleotide (nt) 1,543–1,570. The bottom lines represent the two different *RNF43* frameshifts acquired after CRISPR/Cas9 modulation; *p.V520fs* (−2 nt) and *p.D516fs* (−8 nt).

C Fluorescence images of smFISH showing individual RNF43 mRNA dots in WT SW480 cells and cells carrying mutated *RNF43* alleles. DAPI (blue) is used for nuclear staining. Scale bar represents 10 μm.

D Graph indicating the number of mRNAs for RNF43 per cell for the indicated conditions. Black line indicates mean mRNAs per cell for n = 77 (WT) and n = 41 (*p.V520fs/p.D516fs*) cells.

Data information: WT; wild-type.

© 2020 The Authors

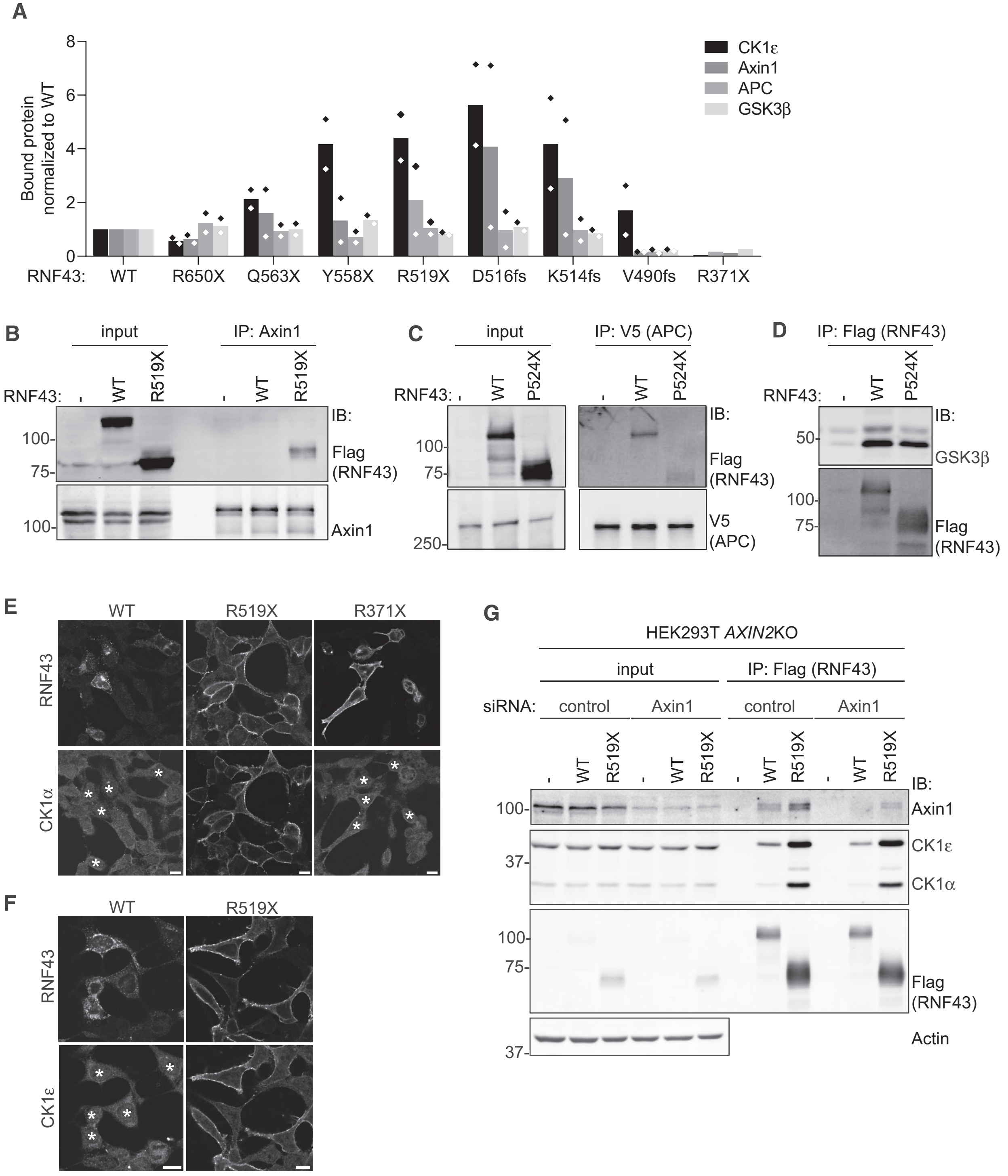

Figure EV3.

 © 2020 The Authors

Figure EV3. Onco-RNF43 binds and relocates the destruction complex proteins Axin1, CK1α, and CK1ε to the plasma membrane.

A Quantification of the Western blot analyses of endogenous destruction complex components co-precipitating with the indicated RNF43 cancer variants shown in Fig 3B. Values were normalized to the amount of protein bound to RNF43 WT. Diamonds indicate the normalized values of the replicates.
B Western blot analysis of RNF43 WT and oncogenic RNF43 (R519X) co-precipitated with endogenous Axin1 in HEK293T cells.
C Western blot analysis of RNF43 WT and oncogenic RNF43 (P524X) co-precipitated with V5-APC in HEK293T cells.
D Western blot analysis of myc-GSK3β co-precipitated with RNF43 WT and oncogenic RNF43 (P524X) in HEK293T cells.
E Confocal microscopy analysis of subcellular endogenous CK1α localization upon expression of RNF43 WT, oncogenic RNF43 (R519X) or R371X. RNF43 proteins are visualized by Flag staining. Asterisks indicate RNF43-expressing cells. Scale bars represent 10 μm.
F Confocal microscopy analysis of the subcellular endogenous CK1ε localization upon expression of RNF43 WT and oncogenic RNF43 (R519X). RNF43 proteins are visualized by Flag staining. Asterisks indicate RNF43-expressing cells. Scale bars represent 10 μm.
G Western blot analysis of endogenous Axin1, CK1ε, and CK1α co-precipitated with RNF43 WT and oncogenic RNF43 (R519X) expressed in *Axin2*KO HEK293T cells treated with control or Axin1 siRNA for 4 days.

Figure EV4. Regulation of RNF43 WT and R519X by CK1 binding and phosphorylation.

A Western blot analysis of endogenous CK1ε co-precipitated with the indicated RNF43 variants expressed in HEK293T cells.
B Mapping the CK1 interaction region in oncogenic RNF43 (R519X) using triple alanine scanning. Western blot analysis of endogenous CK1α and CK1ε that co-precipitated with the indicated oncogenic RNF43 triple alanine mutants expressed in HEK293T cells.
C β-catenin-mediated reporter activity in HEK293T cells induced by the oncogenic RNF43 (R519X) triple alanine mutants used in (B). Average β-catenin-mediated reporter activities ± s.d. in n = 2 independent wells are shown.
D, E β-catenin-mediated reporter activity in HEK293T cells expressing the indicated RNF43 mutants in the presence and absence of Wnt3a. Average β-catenin-mediated reporter activities ± s.d. in n = 2 independent wells are shown.
F Western blot analysis of endogenous CK1α and CK1ε co-precipitated with the indicated RNF43 variants expressed in HEK293T cells.
G β-catenin-mediated reporter activity in HEK293T cells expressing full-length RNF43 WT, ALAA, DLDD, or ΔS486-G489>R mutants. Cells were treated with control medium (no Wnt3a) or Wnt3a-conditioned medium (Wnt3a) overnight. Average β-catenin-mediated reporter activities ± s.d. in n = 2 independent wells are shown.
H Western blot analysis showing the effect of the indicated RNF43 variants on V5-FZD5 expression in HEK293T cells. Open and closed arrows indicate mature (post-Golgi) and immature (ER-associated) FZD5, respectively.

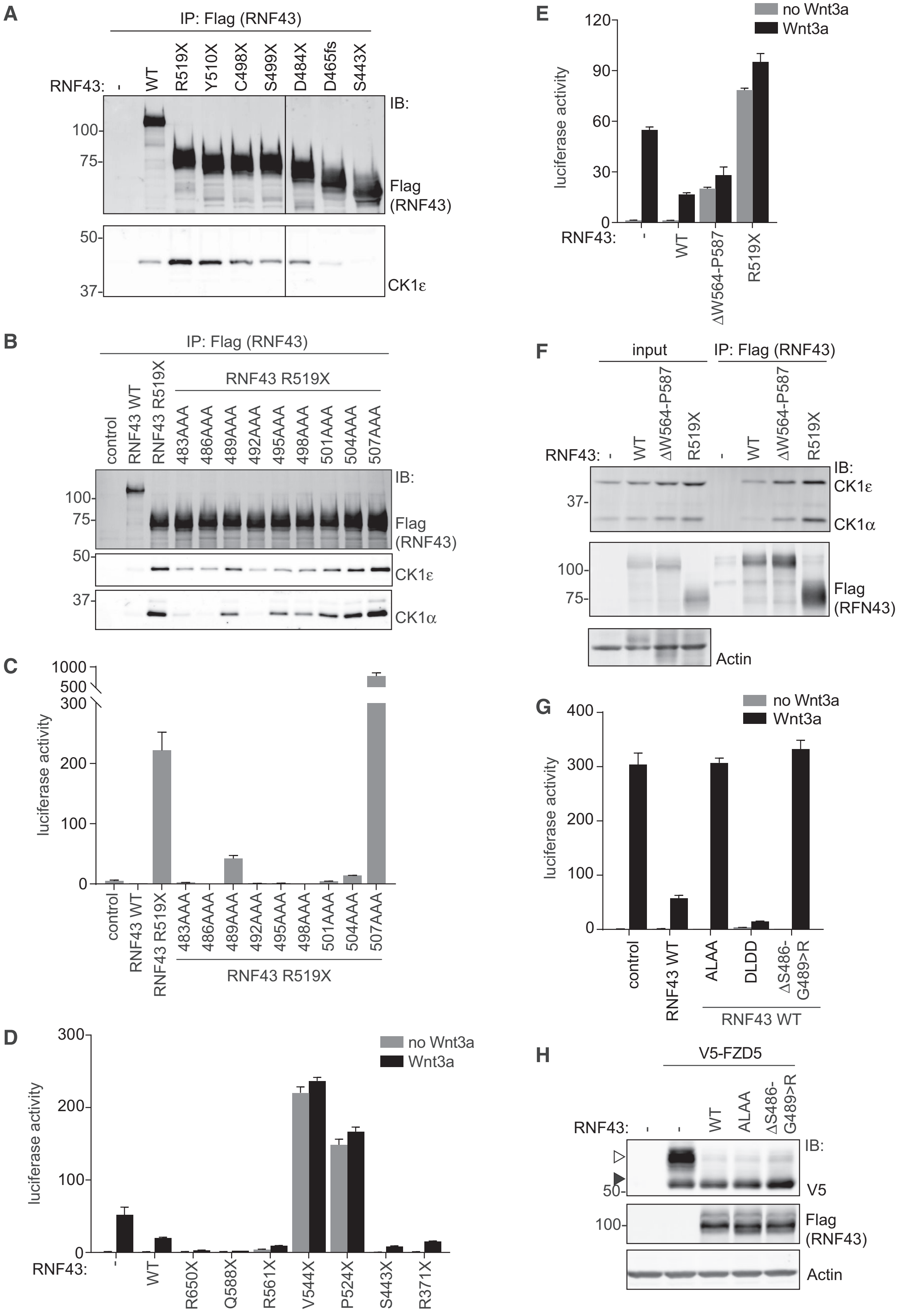

Figure EV4.

 © 2020 The Authors

Figure EV5. Comparison of onco-*RNF43* induced transcriptional alterations in human colon organoids grown in high Wnt/high Rspo and no Wnt/low Rspo conditions.

A Heatmap showing gene expression dynamics for genes that are significantly changed between onco-*RNF43/TP53*KO and *TP53*KO (FDR < 0.01). Relative changes in gene expression are shown as row Z-scores. The first heatmap shows the dynamics between samples grown in no Wnt/low Rspo (0.2%) medium. Rows in the second heatmap are matched to the first heatmap and show gene expression dynamics in organoids grown in full medium. The right heatmap (green/purple) shows the $\log_2$ fold change between all samples in full medium compared to all samples in medium with no Wnt/low Rspo (0.2%). Hierarchical clustering and k-means clustering was performed on row Z-scores in the first heatmap and used to identify four clusters of genes with distinct expression dynamics between the organoid lines.

B UpSet plot showing the number of significantly changing genes in different comparisons. Pairwise comparisons between WT, *TP53*KO, and onco-*RNF43/TP53*KO organoids were performed on growth medium with high Wnt/Rspo (20%) (green) and without Wnt/low Rspo (0.2%) (blue). In addition, all samples in high Wnt/Rspo were compared to all samples in medium with no Wnt/low Rspo (black). Number of significantly changing genes (FDR < 0.01) in each comparison is shown in the horizontal bar plot. Intersections of genes that change significantly in more than one comparison are shown as connected dots in the UpSet plot. The number of genes in each intersection is shown in the vertical bar plot. Only the 50 biggest intersection groups are shown. Multiple testing correction was done with the Benjamini–Hochberg procedure on all pairwise comparisons combined.

© 2020 The Authors

A

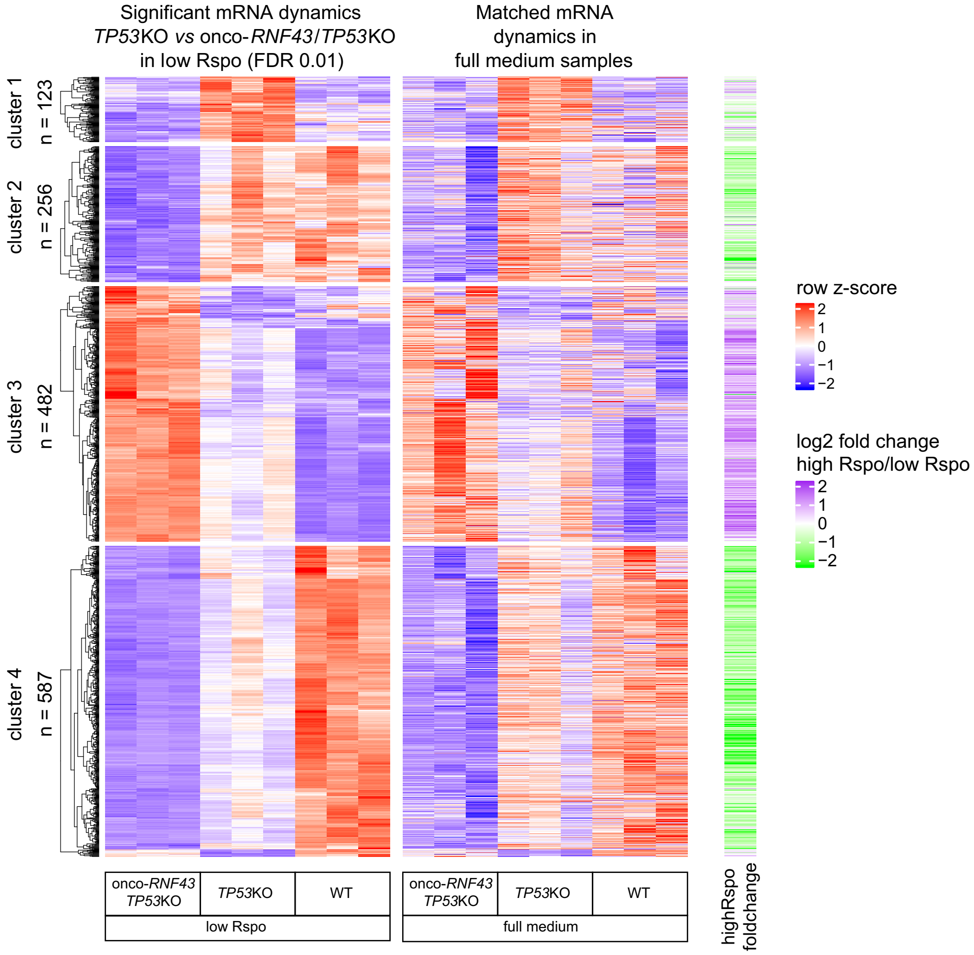

B

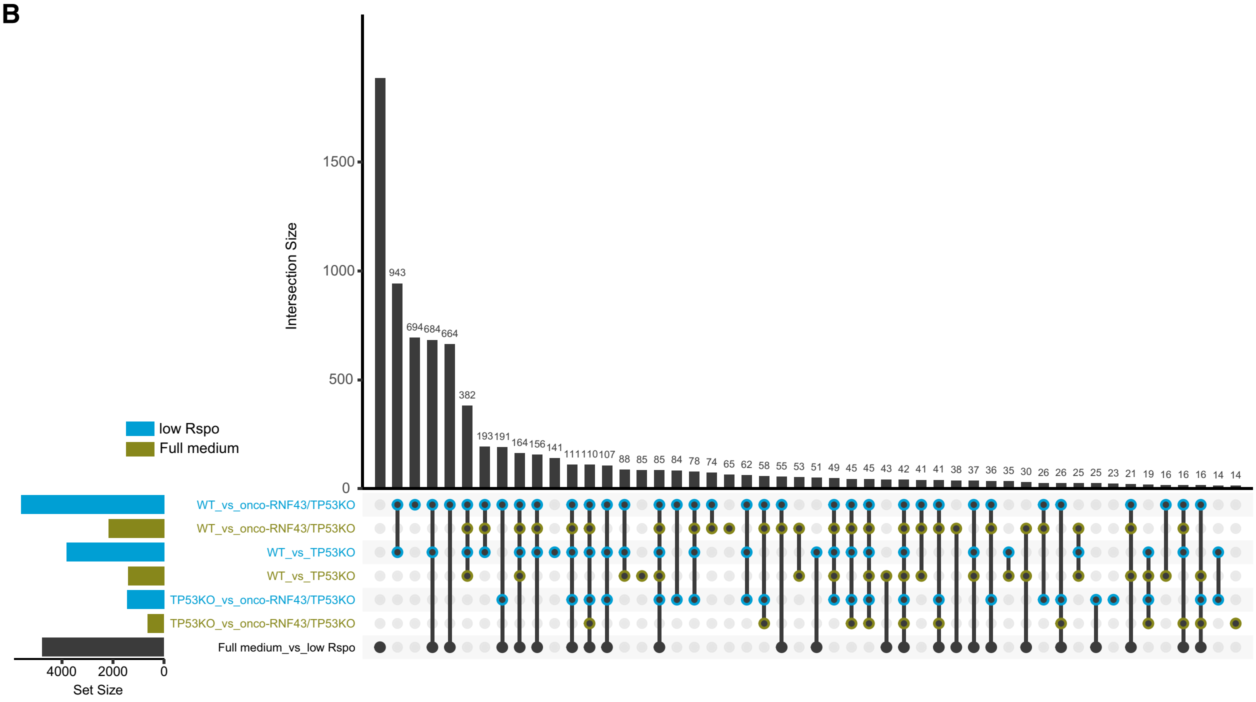

Figure EV5.

© 2020 The Authors
